# The Prevalence and Awareness of Dietary Supplement Use Among Saudi Women Visiting Fitness Centers in Riyadh, Saudi Arabia

**DOI:** 10.7759/cureus.41031

**Published:** 2023-06-27

**Authors:** Shahad Aljebeli, Reem Albuhairan, Nouf Ababtain, Taima Almazroa, Saeed Alqahtani, Winnie Philip

**Affiliations:** 1 College of Medicine, King Saud Bin Abdulaziz University for Health Sciences (KSAU-HS), Riyadh, SAU; 2 Department of Family and Community Medicine, Ministry of National Guard - Health Affairs, Riyadh, SAU; 3 College of Applied Medical Sciences, King Saud Bin Abdulaziz University for Health Sciences (KSAU-HS), Riyadh, SAU

**Keywords:** supplements, physical fitness, saudi women, vitamin, gym-users, gym, nutrition, sports facilities, saudi arabia, dietary supplements

## Abstract

Background

Dietary supplements are used for a variety of reasons, some of which are for medical conditions, to compensate for dietary insufficiency, to improve physical performance, and to boost immunity. Generally, individuals who visit sports facilities seek different benefits; it could be for health and well-being, to achieve the desired body shape, for enjoyment purposes, or as a way to socialize. To help achieve some of these goals, dietary supplements can be taken.

Aims

This study is designed to assess the prevalence and awareness of dietary supplement use among Saudi women who frequent fitness centers in Riyadh, Saudi Arabia.

Methods

A cross-sectional study was conducted in November 2021 among Saudi women who visited gyms in Riyadh, Saudi Arabia. The sample consisted of 355 participants, all of whom were women from Saudi Arabia. The statistical analysis was done using IBM Statistical Package for Social Sciences (SPSS), Version 21.0 (Armonk, NY: IBM Corp.). Necessary statistical tests such as the chi-square, the t-test, and other appropriate tests were used. A p-value of less than 0.05 has been adopted for statistical significance.

Results

The majority of the 355 female participants consumed dietary supplements (68.7%). The most commonly used supplements were vitamins (82.8%), amino acids and proteins (30.3%), minerals (22.1%), and fatty acids (3.3%). In the study group, 53.3% of those who were using or used supplements had previous knowledge; 13.1% were up-to-date; and 33.6% weren’t. No relationship was found between supplement use and educational level, weight, body mass index (BMI), or marital status.

Conclusion

Dietary supplement use is common among women who visited gyms in Saudi Arabia. Vitamins were the most commonly used supplements, which suggests that users were more concerned about general health and diet deficiencies than anything else. The participants use dietary supplements cautiously; the majority consume dietary supplements under a doctor’s prescription and after reading the leaflets.

## Introduction

Dietary supplements (DSs), synthetic or derived from natural resources, are categorized as micronutrients, which include minerals, vitamins, and macronutrients such as amino acids, fatty acids, and carbohydrates [1]. Dietary supplements are used for a variety of reasons, some of which are for medical conditions, to compensate for dietary insufficiency, to improve physical performance, and to boost immunity [1]. Generally, individuals who visit sports facilities pursue different benefits, including health and well-being, to achieve the desired body shape, for enjoyment, or as a way to socialize [2]. Dietary supplements are taken to support the acquisition of some of these goals. [3]. DSs have proven to be effective, as 69.4% of the male gym members in Riyadh reported that the use of supplements supported the achievement of their body goals [3]. Dietary supplements have great benefits; they are also being used by US military programs to increase muscle mass and build the body to maintain a specific standard of body composition [4]. However, dietary supplements may cause side effects if taken without medical supervision, as high doses can cause serious complications. As an example, the consumption of pyridoxine (vitamin B6) in high doses is associated with neurotoxicity and photosensitivity [5].

Based on current evidence, there is a clear lack of research exploring the prevalence of using dietary supplements among women who visit sports facilities. This research aimed to investigate the prevalence and awareness of using dietary supplements among female gym attendees in the Riyadh region of Saudi Arabia.

## Materials and methods

Study design, area, and settings

The study was conducted from November 2021 until January 2022. It was a cross-sectional, questionnaire-based study. The participants were women ≥18 years of age who visited fitness centers in Riyadh, Saudi Arabia. The Ministry of Sports in Riyadh, Saudi Arabia, provided a list of all fitness centers, and 25 were randomly selected using a randomized cluster sampling technique.

Identification of study participants

The study was conducted on 355 women who visited the fitness centers; all the participants were Saudis. The required sample size was calculated using the OpenEpi epidemiologic calculator (Open Source Epidemiologic Statistics for Public Health, Version, www.OpenEpi.com, updated 2013/04/06) based on 50% prevalence. The population size estimate was acquired from The General Authority of Statistics in Saudi Arabia. Using a 95% confidence interval and a margin of error of +5, a nonrandom, convenience sampling technique was used to obtain the sample of participants.

All women, aged 18 years and older, who visited the chosen fitness centers were included. Women aged less than 18 years or more than 64 years of age and non-Saudis were excluded from this study.

Data collection process

The data were collected using a self-administered questionnaire, which was distributed by visiting the fitness centers as well as via an online link (Appendix 1). The data collection was done over a period of three months. The questionnaire was adapted from a validated questionnaire that included 19 items divided into four parts. The first part (questions one to five) included marital status, level of education, weight, age, and gender. The second part (questions six to nine) measured the knowledge and prevalence of the consumers (e.g., the relevant knowledge about supplements, personal consumption, and type of dietary supplements used). The third part (question 10) was used to test the relationship between the consumption of dietary supplements and knowledge regarding them. The fourth part (questions 11-19) was used to demonstrate the attitude of the consumers about the dietary supplements consumed (e.g., knowledge of benefits and side effects of the dietary supplements used, advice and usage of dietary supplements, purchase locations, and money spent on supplements).

Data analysis

The data were entered and analyzed using IBM Statistical Package for Social Sciences (SPSS) for Windows, Version 21.0 (Armonk, NY: IBM Corp.). Descriptive statistics were expressed in the form of frequency and percentage for the baseline demographic characteristics (e.g., gender, marital status, knowledge level, attitude). In the analytic statistics, Pearson’s chi-square (χ2) was used to test the association between the categorical variables, including the use of dietary supplements and knowledge, age, level of education, weight, and height. The statistical significance was set at a p-value<0.05.

Informed consent was obtained from the participants. They were given full anonymity because the questionnaire did not include any personal data that could identify them. Ethical approval was obtained from the Institutional Review Board (IRB) of the King Abdullah International Medical Research Center (KAIMRC), Riyadh, Saudi Arabia (Approval number: RYD-21-419812-74276).

## Results

The study included 355 participants to determine the prevalence and awareness of the use of dietary supplements among women who visit gyms in Riyadh, Saudi Arabia. All the participants were provided with a self-administered questionnaire. The demographic characteristics of the sample are illustrated in Table 1.

**Table 1:** Demographic characteristics of study subjects

Variable	Number (Percentage)
Age (in years)	
18-25	233 (65.6)
26-33	75 (21.1)
>=34	47 (13.2)
Marital status	
Single	276 (77.7)
Married	69 (19.4)
Divorced	10 (2.8)
Education	
General education (high school or less)	97 (27.3)
High education (more than high school)	258 (72.6)
BMI	
Underweight	35 (9.8)
Normal	211 (59.43)
Overweight	67 (18.87)
Obese	42 (11.83)
Total	355(100)

The majority (65.6%, n=233) of the participants were 18-25 years old, and most of the participants were single. In terms of the level of education, the majority had higher education (higher than high school) (72.6%) and 27.3% general education (high school or lower). In terms of BMI, the majority had a normal BMI (59.43%), 18.87% were overweight, 11.83% were obese, and 9.8% were underweight.

The majority of the participants (68.7%) consumed dietary supplements, and in the supplement use group, 53.6% had some knowledge, 13.1% were up-to-date, and 33.6% were not knowledgeable regarding supplements. The main reason underpinning the use of the supplements was as a treatment (64.7%), followed by support in the gym (40.2%). Regarding the participants' views about DSs, most indicated that it improved a diet deficiency (72.1%), provided nutrients faster (19.3%), gave the body all it required (12.2%), helped with a diet deficiency, provided faster nutrition, and gave the body all the required nutrients (9.8%), Among the study group, 1.6% thought that dietary supplements didn't help. The participants perceived that the consumption of dietary supplements should be based on a doctor’s prescription (78.7%), a personal decision (13.1%), a coach's advice (6.6%), or a friend’s advice (1.6%). Only 52.9% of the participants read the leaflets before consuming the supplements, and 28.3% sometimes read them; however, 18.9% did not read them at all. The majority (70.9%) indicated that it should be used under a doctor’s prescription, 27% said the diet should provide all one’s nutrients, and 2% warned against using it. (Table 2).

**Table 2:** Participants' views on the use of dietary supplements and the factors affecting usage

Particulars	Number (Percentage)
Do you consume dietary supplements and multivitamins now or have you, in the past?	
Yes	244 (68.7)
No	111 (31.3)
Total	355 (100.0)
If yes, are you up-to-date with dietary supplements and multivitamins?	
Yes	32 (13.1)
No	82 (33.6)
Have some knowledge	130 (53.3)
Total	244 (100.0)
Dietary supplements and multivitamins help in	
Work	25 (10.2)
Gym	98 (40.2)
Assist in treatment	114 (64.7)
All	32 (13.1)
Do not help	24 (9.8)
Supplements help in	
Giving the body all the nutritional necessities	30 (12.2)
Covering deficit in the diet	176 (72.1)
Giving faster nutrition than a normal diet	47 (19.3)
All	24 (9.8)
Do not help	4 (1.6)
Consuming dietary supplements and multivitamins	
Is not harmful to health	13 (5.3)
is harmful if consumed excessively	216 (88.5)
Does not cause harm or benefit	7 (2.9)
Is harmful to health	8 (3.3)
Consumption of dietary supplements and multivitamins should be	
With a doctor's prescription	192 (78.7)
Based on advice from a sport coach	16(6.6)
Based on advice from a friend	4(1.6)
An own decision (self-acting)	32(13.1)
Do you read the leaflet that comes with dietary supplements and multivitamins?	
Yes	129 (52.9)
No	46 (18.9)
Sometimes	69 (28.3)
Do you advise the usage of dietary supplements and multivitamins?	
Yes, with a medical prescription	173 (70.9)
Believe that diet covers all the nutrients needed	66 (27.0)
Warn against the usage of supplements	5 (2.0)

**Figure 1:**
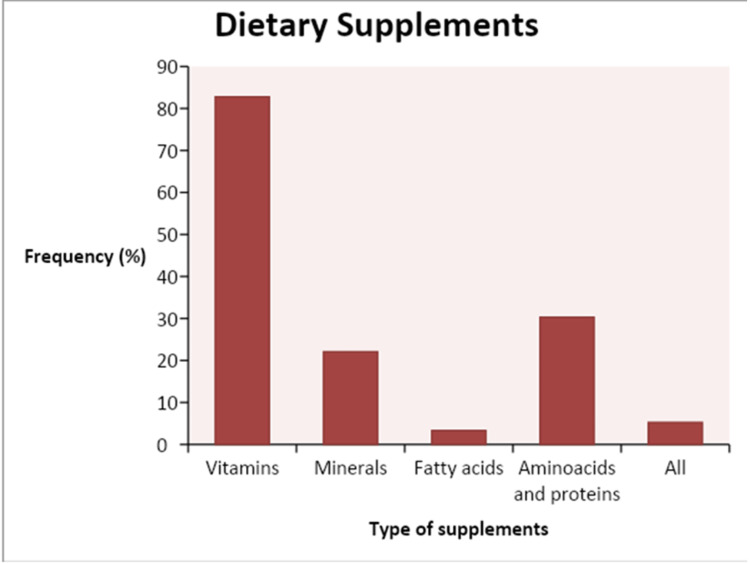
Type of dietary supplements used by the participants

As seen in Figure 1, the most frequently used supplements were vitamins (82.8%), amino acids and proteins (30.3%), minerals (22.1%), and fatty acids (3.3%). In the study group, 4.1% used all of them.

**Figure 2:**
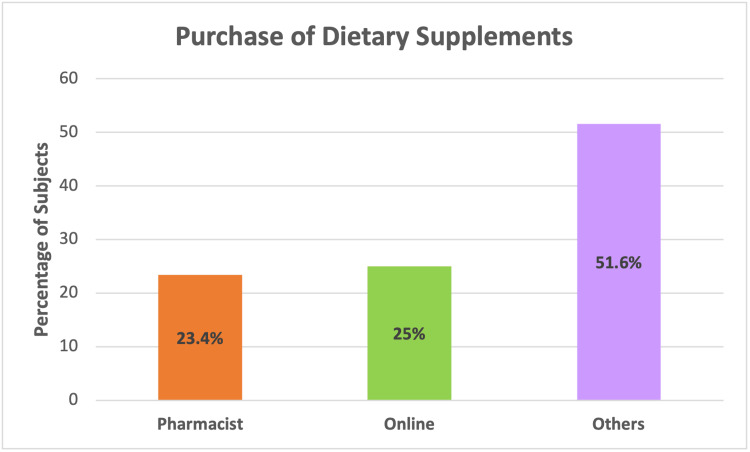
Sources for purchase of dietary supplements

As displayed in Figure 2, the participants were mostly buying dietary supplements from pharmacists (23.4%), online (25%), and other ways (51.6%).

**Figure 3:**
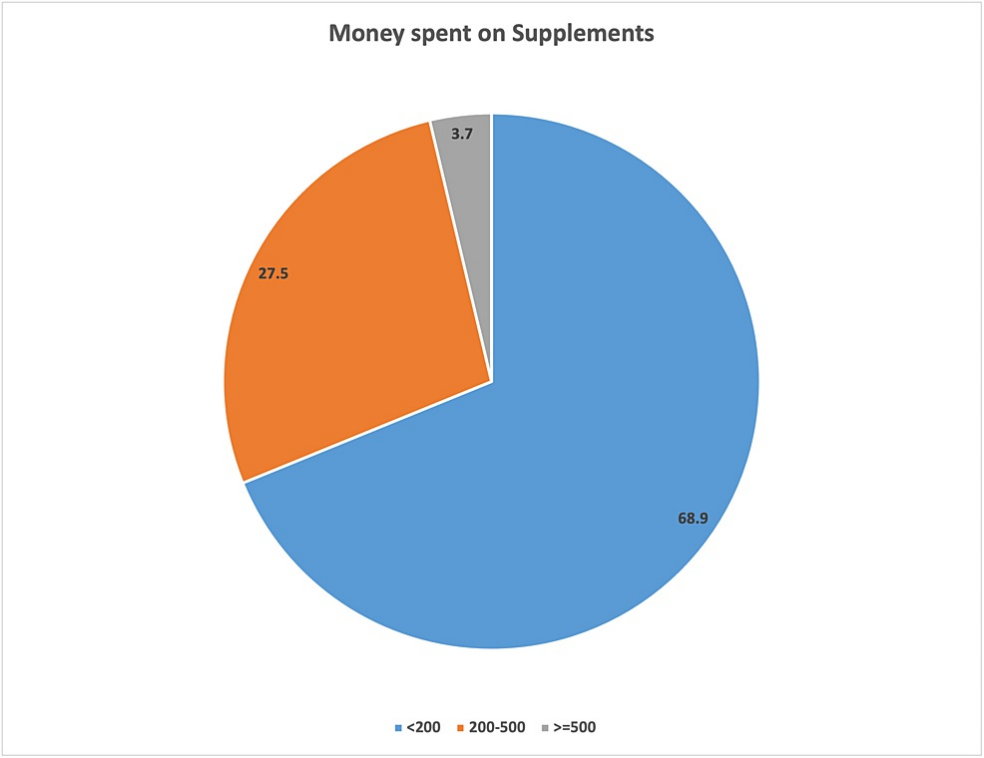
Pie diagram for money spent on supplements (in percent) <200; 200-500; >500: money spent in SAR

For the majority (68.9%) of the participants, as illustrated in Figure 3, the supplements they used cost them less than 200 Saudi riyals (SAR) per month, and 27.5% spent 200 to 500 SAR per month; only 3.7% of the participants spent more than 500 SAR per month.

Regarding the relationship between demographic characteristics and the use of supplements, Table 3 indicated no statistically significant difference between the use of supplements and age, marital status, BMI, or education (p-value>0.05). It was noticed that most of the participants (n=244, 68.7%), regardless of their demographic characteristics, have used or are using dietary supplements.

**Table 3:** Characteristics of dietary supplement use based on demographic variables

Use of supplements no. (%)				p-value
Variable	Yes	No	Total	
Age (in years)				
18-25	161 (69.1)	72 (30.9)	233 (100)	0.979
26-33	51 (68)	24 (32)	75 (100)	
>=34	32 (68.1)	15 (31.9)	47 (100)	
Marital status				
Single	188 (68.1)	88 (31.9)	276 (100)	0.404
Married	47 (68.1)	22 (31.9)	69 (100)	
Divorced	9 (90)	1 (10)	10 (100)	
Educational qualification				
General education	61 (62.9)	36 (37.1)	97 (100)	0.145
High education	183 (70.9)	75 (29.1)	258 (100)	
BMI (kg/m sq)				
Underweight	22 (62.9)	13 (37.1)	35 (100)	0.117
Normal	151 (71.6)	60 (28.4)	211 (100)	
Overweight	39 (58.2)	28 (41.8)	67 (100)	
Obese	32 (76.2)	10 (23.8)	42 (100)	
Total	244(68.7)	111(31.3)	355(100)	

## Discussion

Although there is no conclusive proof of whether dietary supplements are beneficial or harmful, they are widely distributed on the market and are frequently used by athletes and individuals who are interested in following a healthy lifestyle [6]. More than 90% of Saudi athletes use different kinds of dietary supplements; however, most users do not gain enough information before starting to use them [7,8]. The main resources for obtaining information about DSs are the Internet, fitness coaches, and physicians, respectively [3]. The findings of a study in Riyadh showed that 58% of the participants were using supplements under professional supervision; additionally, weight loss and bodybuilding were the leading motives for taking the supplements [3]. Proteins were the most frequently used supplement (29%), followed by multivitamins (21%) [3]. Another study conducted in Riyadh reported that for 48% of people visiting gyms who were using nutritional supplements, protein powder was the most frequently used supplement. In addition to that, friends were the main source of information about the supplements (40%) [8]. A study done in Taif, Saudi Arabia, with gym attendees indicated that only 29.8% of the participants used DSs, which is low compared with the studies in Riyadh. A similarity was that proteins were also the most commonly used supplement (22.5%) [9].

Internationally, a study done in Portugal with adult athletes reported that the supplements used most were proteins (80.1%), multivitamins and/or minerals (38.3%), and sports bars (73.3%) [10]. The motives underlying the use of supplements were building muscles (55.7%), speeding up recovery (52.7%), and improving performance (47.3%) [10]. More than 70% of gym members were well-informed about supplements, with only 4% being uninformed about them [10]. In the UAE, protein supplements were also the most frequently used dietary supplements, followed by multivitamins [11]. The main source of information regarding using supplements in the UAE was the Internet (60.7%), and only 12.8% of the individuals had prescriptions from nutritionists [11]. In contrast to the literature, a study done in Germany with adolescent athletes indicated that the most commonly used supplements were minerals (87%), vitamins (76%), and protein (30%) [12]. The athletes were taking dietary supplements as advised by their parents (34%), physicians' prescriptions (24%), or independently (7%) [12]. However, only 36% of German users knew about the side effects of supplements [12].

There has also been an increase in supplement use in recent years. The most important factor was the rising trend in advertisements by unauthorized figures on social media presenting false impressions or ideas regarding how essential and beneficial supplements could be. A cross-sectional study done in the Netherlands showed a negative relationship between image-centric social media use and body image in young male gym users. The current study expands on previous findings by specifically showing that image-centric social media such as Instagram and exposure to fitness-related content are associated with body dissatisfaction and with the use of dietary supplements and anabolic androgenic steroids [13].

There is no mention of when a supplement is truly required, as they should only be used to correct a diet deficiency and not be taken without probable cause [14,15]. A study that aimed to investigate the use of dietary supplements in patients in Japan concluded that most patients used dietary supplements without consulting physicians, and some of them experienced adverse effects from using dietary supplements. To avoid health problems, it is important that physicians ask patients about dietary supplement use, and those patients should inform their physicians about these supplements if physicians do not ask [16]. For example, iron supplements are advertised as a necessity, especially for women since the rates of iron deficiency anemia in women are high, without informing them that minerals such as iron can be toxic to the body since they do not have an efficient mechanism for excretion [17-19].

During the COVID-19 pandemic, many people became concerned about their health and well-being, which led them to augment their diet with supplements [20, 21, 22]. Dietary supplement sales have dramatically risen during the COVID-19 pandemic despite depressed economic conditions. Common DSs used were immune-modulating dietary supplements, including vitamin D, ascorbic acid, zinc, and melatonin [23]. This study indicated that of the 355 participants, the majority (68.7%) did consume dietary supplements, with only 13.1% being knowledgeable about the benefits and adverse effects. The consumption of dietary supplements could be due to multiple reasons, one of which is the social media effect and rising trends. Second, dietary supplements are easily accessible. In addition, they are used to maintain overall health and well-being [24]. The majority of participants have in-depth knowledge of the supplements they consume. The reason for this might be the quick access they have to informative websites.

In this study, the main reason behind the use of the supplements was as a treatment (64.7%), due to the recommendation of their health providers, as seen in Table 2. The majority (78.7%) agreed that supplements should be consumed following their health providers' prescriptions and/or recommendations. Most participants believed that supplements should be used to cover any diet deficiency, as supported by Schuetz et al., who reported that a large number of participants (54.41%) agreed on the fact that dietary supplements are taken to aid in a deficiency in the diet [22]. However, it was not clear what their standards or definition of a deficient diet are; they can differ from unrealistic standards due to the influence of social media and/or society, as opposed to Bailey et al.'s study, which showed the majority of the participants (45%) were consuming DSs to improve overall health, followed by "bone health" in 25% of the participants [25].

An interesting finding in Knapik et al.'s meta-analysis showed that when compared to the prevalence of the use of DSs between elite athletes and non-elite athletes, elite athletes tended to use dietary supplements to a greater extent than non-elite athletes [26]. Maughan et al. explained this by giving the reasons why elite athletes consume more DS in order to aid in recovery from training and improve health and/or performance [27].

When asked if supplement use is advised, 70.9% believe it should be used with a doctor’s prescription, which can explain why the majority perceived DS as a treatment; 27% indicated that a person’s diet should include all the required nutrients, and 2% warned against using DS, as supported by Algaeed et al. [14]. A possible reason is an increase in awareness of diet and supplement use due to the availability of data [28].

The results of the study shown in Figure 1 showed that vitamins were the most frequently used supplements by the participants. Similar findings were reported by Maughan et al.. The most commonly used supplements were vitamins and antioxidants, which were consumed by 84% of their participants [27]. This is contrary to the findings of [8, 26, 29], who reported that the most frequently used supplements were proteins. This might be explained by the fact that the participants of the current study were women, in contrast to their samples being both men and women. The current sample was not as interested in building muscles as compared to men.

The participants were mostly purchasing DSs from pharmacies or online. The majority of the participants used dietary supplements under a physician’s prescription, easily obtained from a pharmacy. The financial status of the consumers was not an important factor in the purchase of supplements, as the majority spent less than 200 SAR per month.

The current study found no significant associations between the use of supplements and age, marital status, BMI, or education (p-value>0.05). This is in contrast to a study that was done in Saudi Arabia with male athletes, which indicated a significant relationship between age, BMI, and the use of supplements [3]. In the current study, the participants were using supplements regardless of their weight, age, or educational level.

## Conclusions

Dietary supplements are frequently used by women who visit gyms in Saudi Arabia. Vitamins were the most commonly used supplements, which suggests that the users were more concerned about their general health and diet deficiencies than anything else. The participants used dietary supplements cautiously; the majority consumed dietary supplements under a doctor’s prescription and after reading the leaflets. Due to a lack of research regarding the use of supplements by Saudi women who visit gyms, we recommend conducting more studies with a larger sample size.

## Appendices

Appendix one

Questionnaires distributed to participants in the study

**Table 4:** Relationship between supplement consumption and a few variables

Marital status
Single
Married
Divorced
Level of education
Elementary school
Middle school
High school
Bachelor’s degree
Higher education
Weight
Below average BMI
Normal BMI
Above average BMI
Age
18-25 years old
26-33 years old
34-41 years old
42 years and above
Gender
Male
Female

**Table 5:** Participants' knowledge of supplements and their prevalence

Are you up-to-date with dietary supplements and multivitamins?
Yes
Have some knowledge
No
Do you consume dietary supplements and multivitamins now or have you consumed them in the past?
Yes
No
If not, do you know anyone who consumes it? (n=250)
A family member
A friend
Do not know
Type of dietary supplements used
Vitamins and multivitamins
Minerals
Fatty acids
Amino acids and proteins

**Table 6:** Relationship between consumption and knowledge of DSs

Are you up-to-date with dietary supplements and multivitamins?
Yes
I have some knowledge
No

**Table 7:** Characteristics of the participants' attitude towards supplements

Dietary supplements and multivitamins help in
Work
Gym
Assist in treatment
Do not help
Supplements help in
Giving the body all the nutritional necessities
Covering any deficit in the diet
Giving faster nutrition that a a normal diet
Do not help
Consuming dietary supplements and multivitamins
Is not harmful to health
Is harmful if consumed excessively
It does not cause harm or benefit
is harmful to health
Consumption of dietary supplements and multivitamins should be
With a doctor's prescription
Based on advice from a sports coach
Based on advice from a friend
Own decision (self-acting)
Do you read the leaflet that comes with dietary supplements and multivitamins?
Yes
No
Sometimes
Do you advise the usage of dietary supplements and multivitamins?
Yes, with a medical prescription
Believe that diet covers all the nutrients needed
Warn against the usage of supplements
Purchase of dietary supplements and multivitamins through
Pharmacists
Internet
Others like (specialized shops, friends, gym, hospital, etc.)
Money spent on supplements
Less than 200 SAR(50 USD)
Between 200 and 500 SAR (50 to 130 USD)
More than 500 SAR (above 130 USD)
The longest period of time you have gone without eating fresh fruits and vegetables
Less than a week
A week
Two weeks
More than two weeks
